# The Streptococcus Epidemic at Camp Taylor, Ky.

**Published:** 1918-11

**Authors:** 


***The Streptococcus Epidemic at Camp Taylor, Ky.*** By HERBERT FOX, and WALTER W. HAMBURGER. *The Journal of the American Medical Association,* June 8, 1918.

From investigations conducted at Camp Taylor, Ky., it was surprising to see that a very great proportion of the morbidity in the camp was to be attributed to streptococcus infections, bearing not only on the respiratory tract but on practically every living tissue of the body.

Measles were not the chief cause of death, but broncho-pneumonia, assuming the usual symptoms of measles.

In the almost pandemic infection the men were seized with the disease very rapidly, were completely prostrated, and empyema was soon detected; the lesions, from catarrhal, became hemorrhagic, and there was evidence of septicemia. Some of the cases developed erysipelas and steptoccoccus cellulitis.

The multiplicity of lesions revealed at necropsy brought forcibly the proof that streptococcus infection was a separate infection from measles. In ante-mortem examinations it was also found that in some cases where measles subsided, the streptococcus increased in virulence; but it is also true that measles have been increased in severity by the presence of a streptococcus.

The origin of the infection is probably to be traced to a local process or to a primary septicemia, according to cases; spread to the pleura can be lymphogenic or peribronchial, or brought through the blood stream. Primary empyema must be due to hematogenic spread.

The rise of virulence at Camp Taylor in the autumn of 1917 is explained thus : the considerable wind and dust caused a great deal of coughing, increasing the possibilities of infection tremendously. The men who had come from rural districts were the most affected. Some had arrived at the camp already infected, harboring hemolytic steptococci in the pharynx.

Life in camp certainly propagates infection. A single company of men at camp for six months showed as many as 83 o/o carriers of hemolytic streptococci in the pharynx, when examination on entrance to the camp gave 15 o/o carriers of hemolytic streptococci in the pharynx.

This increase is due not only to greater opportunities for infection, such as crowded halls or barracks, but also to the fact that the men do not report soon enough and contaminate those about them. It has been suggested that instead of waiting until the men report themselves sick, the medical officers should weed out those he considers a menace to the community. So much for men in camp. Before entering camp the local exemption board might be made to inspect the drafts, even before they board the train.

Once recognized ill, the men should be isolated as much as is possible. The isolation of patients on entering the hospital reduced incidence; washing the floors with disinfectants also improved conditions, but where the use of bactericidal solutions failed, it was seen how promptly the carriers placed out on the porches with abundance of fresh air, good food and exercise, recovered.

The existing conditions of camp life make swabbing and ensuing personal isolation an impracticable arrangement. Vaccination would be of real benefit if cellular immunity was sure to occur. The detention camp affords better prospects of success.

Once in the infirmaries, all coughing patients should wear a mask, and the wards should be divided by sheets into cubicles so as to prevent crossed infection. All attendants of the ward must be masked and gowned, adopting, in a word, the method installed at Camp Grant by Major Capps.

